# A case of recurrent metastatic ameloblastoma and hypercalcaemia successfully treated with carboplatin and paclitaxel: long survival and prolonged stable disease

**DOI:** 10.3332/ecancer.2013.323

**Published:** 2013-06-04

**Authors:** AF Ghiam, A Al Zahrani, R Feld

**Affiliations:** 1 Princess Margaret Hospital, University Health Network, Toronto, Canada; 2 Department of Radiation Oncology, University of Toronto, Canada; 3 King Faisl Specialist Hospital and Research Center, Riyadh, Saudi Arabia; 4 Department of Medical Oncology and Hematology, University of Toronto, Canada

**Keywords:** metastatic ameloblastoma, carboplatin, paclitaxel, survival

## Abstract

We describe a case of recurrent metastatic malignant ameloblastoma to the lungs with hypercalcaemia in a 47-year-old man. The first lung metastasis was resected nine years after the initial primary, and the tumour recurred with extensive pulmonary metastases 21 years after the primary tumour was resected. This case presented with malignancy-associated hypercalcaemia, likely due to paraneoplastic syndrome, which is exceedingly unusual in association with malignant ameloblastoma. He was successfully treated with carboplatin/paclitaxel and showed the longest survival and stable disease, from the diagnosis of recurrent metastasis, recorded as a case report. This regimen is reasonably well tolerated and can be repeated safely.

## Introduction

Ameloblastoma is the second most common odontogenic tumour that mostly involves the mandible and maxilla. It is a slow-growing locally invasive epithelial tumour with a high recurrence rate (50%–72%) and rare metastasis (<2%). Histopathologically, metastasising ameloblastoma shows no specific features different from non-metastatic ameloblastoma, which makes it impossible to predict its clinical behaviour. Metastasis usually follows multiple recurrences and frequently occurs in the lungs (≥75%) and cervical lymph nodes, usually many years after the primary tumour [1–4]. Extensive surgical resection of the primary tumour with intent to cure is the mainstay of treatment for ameloblastoma and is proposed as the best approach to prevent recurrence/metastasis.

Because metastatic ameloblastoma is rare, there is little information in the literature to guide the treatment plan, and the evidence is mainly derived from case reports. Here, we describe a case of mandibular ameloblastoma with recurrent extensive pulmonary metastasis controlled effectively with carboplatin/paclitaxel, achieving long-term partial remission. This case is also interesting because hypercalcaemia is rarely reported in metastasising ameloblastoma, and the absence of other causes suggested a paraneoplastic aetiology.

## Case report

In March 2010, a 47-year-old male presented to the Lung Clinic at Princess Margaret Hospital, University Health Network, Toronto, Canada, with occasional haemoptysis. His past history was significant due to a resection of a left-sided mandibular ameloblastoma with subsequent hip bone graft in 1989. After being asymptomatic for nine years, he had developed a lung metastasis and had undergone a wedge resection of the right-sided solitary pulmonary metastasis in 1998. Subsequently, he was followed for approximately three years with serial chest x-rays and was discharged, being disease-free at that time. He had a recurrence a second time in 2010 when he was symptomatic with occasional haemoptysis from pulmonary metastases.

**Figure 1:**
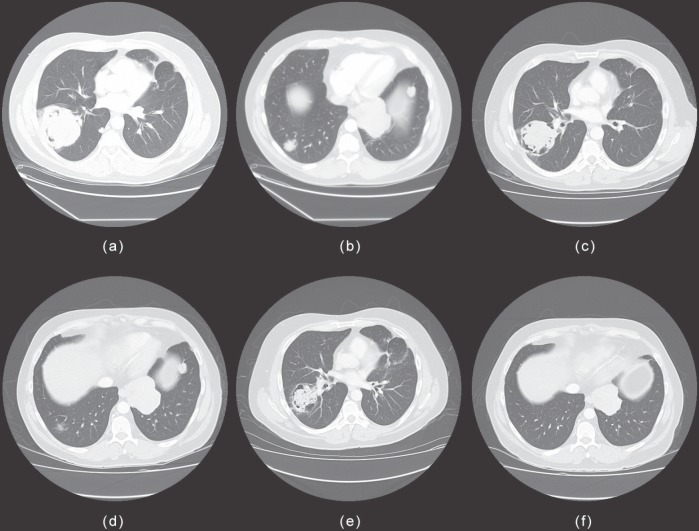
Chest CT scan of the patient treated with carboplatin and paclitaxel: the CT scan showed bilateral intrapulmonary metastases during initial workup with a 7.6 cm × 7.3 cm dominant mass in right lower lobe and a 7.4 cm × 8.2 cm dominant mass in left lower lobe (a,b); partial response after chemotherapy with carboplatin and paclitaxel (c,d); and on latest follow-up, the intrapulmonary masses and nodules had significantly reduced in size or remained stable (e,f). There was no evidence of any enlarging or new intrapulmonary lesions.

A clinical examination was unremarkable, except for alopecia and a scar on the left mandible from the previous tumour resection. The whole-body PET/CT scan and bone scan revealed multiple bilateral pulmonary metastases (Figure 1a,b) with no evidence of extrathoracic disease. The core biopsy was taken from the right lung mass. The pathology showed malignant epithelial neoplasm composed of nests and cords of cells demonstrating nuclear palisading and associated with foci of squamous differentiation. The morphologic features confirmed the diagnosis of metastatic ameloblastoma.

The patient’s situation was also complicated with asymptomatic hypercalcaemia (3.53 mmol/L), likely as part of paraneoplastic syndrome, which was managed initially by intravenous fluids and pamidronate therapy and subsequent therapies administered every 3–4 weeks.

The pulmonary metastases were surgically unresectable and were not amenable to radiotherapy due to the large radiation fields required to encompass the tumours. After a case discussion in our multidisciplinary tumour board, the patient commenced treatment on carboplatin/paclitaxel. Soon after treatment initiation, haemoptysis disappeared, and his calcium was stabilised. A chest radiograph and CT scan taken after three cycles showed disease response (Figure 1c,d), and therefore three additional cycles of the same chemotherapy were given. He successfully completed six cycles with minimal toxicities (Grade 2 peripheral neuropathy and mild nausea/vomiting). Post-treatment, the latest follow-up CT scans demonstrated radiological partial response (Figure 1e,f).

Approximately three years after starting chemotherapy, he is generally feeling quite well (ECOG = 0–1) and is clinically asymptomatic. After a long interval without treatment, his lung disease is stable on follow-up CT imaging and his calcium level is normal. We continue to follow him with intermittent CT scans and would re-institute carboplatin/paclitaxel were there to be a relapse or progressive disease.

## Discussion

Metastatic ameloblastoma is rare, and metastasis is often a strikingly late event after the primary occurance. As a result, the treatment of metastatic ameloblastoma is elusive. There is inadequate evidence in the literature to guide the treatment plan, and treatment decisions are mainly taken from case reports.

For lung metastases, pulmonary metastasectomy with maximum preservation of viable lung tissue remains the mainstay of treatment, and the role of cheomotherapy and/or radiation is yet to be defined. Based on the available data from published case reports, chemotherapy at the physician’s discretion has been the treatment of choice when radical resection could not be achieved. There is no single-agent or combination chemotherapy regimen that can be recommended for palliation in patients with unresectable metastases. Different palliative chemotherapy regimens are reported to have reduced the tumour size and improved symptoms (for review, see reference [3]) [1–5].

With these rare tumour subtypes, chemotherapy options are limited by a lack of experience and variable efficacy as well as non-optimal treatment results. Only very few reports exist in the literature on the use of platinum-based treatment in this indication [3, 5, 6]. Here, we described a case of mandibular ameloblastoma with recurrent extensive pulmonary metastasis controlled effectively with carboplatin/paclitaxel, achieving disease stabilisation and long-term partial remission. To our knowledge, this is the second report of metastatic ameloblastoma to the lung that responded well to carboplatin/paclitaxel and with longer survival than the first, suggesting benefits for systemic treatment in cases with inoperable disease and adding evidence to the literature [5]. The longest survival after diagnosis of metastases (37 years) is reported by Hasim *et al *in a patient with bilateral pulmonary metastases, who interestingly remained asymptomatic and did not require any treatment [7].

A rare feature of our case was that a good clinical response was achieved with chemotherapy only. It highlights the benefit of single modality chemotherapy treatment when surgical resection or radiotherapy is not feasible. This case is also interesting because only a few cases of metastasising ameloblastoma with malignancy-associated hypercalcaemia have been reported [3, 8–10]. In the absence of other causes (e.g., osteolytic bone metastasis), it is very likely that the hypercalcaemia is malignancy-associated, with either an elevation in parathyroid hormone-related protein (PTHrP) or other cytokines.

## Conclusion

This report may help to guide physicians to choose a systemic treatment for patients with metastatic and/or disseminated ameloblastoma. This regimen is reasonably safe, well tolerated, easy to administer, and, more importantly, can be repeated, as being used in ovarian cancer. The efficacy for this particular regimen warrants further reports from similar cases. However, in the absence of any proven effective chemotherapy and due to perceived poor prognosis of metastatic ameloblastoma, systemic chemotherapy using carboplatin plus paclitaxel should be considered a treatment option.

## Conflicts of interest

The authors declare no conflicts of interest for this manuscript.
